# Supplementary material for: Population isolation in the Plains spadefoot toad: causes and conservation implications
Source: PeerJ. 2024 Oct 7;12:e17968. doi: 10.7717/peerj.17968 (PMC11466216; doi:10.7717/peerj.17968)
Supplement: Supplemental Information 1 — Museum catalog abbreviations: OMNH: Sam Noble Oklahoma Museum of Natural History, University of Oklahoma; FHSM: Sternberg Museum of Natural History, Fort Hays State University; MVZ: Museum of Vertebrate Zoology, University of California, Berkeley; TNHC: Texas Science & Natural History Museum, University of Texas at Austin; UTA A, Amphibian and Reptile Diversity Research Center, University of Texas at Arlington. [file peerj-12-17968-s001.docx]

Supplemental Table 1. *Spea bombifrons* population groups for the population genetic dataset, including collection location information, sample size, any museum catalog numbers, and GenBank Accession numbers. Museum catalog abbreviations: OMNH: Sam Noble Oklahoma Museum of Natural History, University of Oklahoma; FHSM: Sternberg Museum of Natural History, Fort Hays State University; MVZ: Museum of Vertebrate Zoology, University of California, Berkeley; TNHC: Texas Science & Natural History Museum, University of Texas at Austin; UTA A, Amphibian and Reptile Diversity Research Center, University of Texas at Arlington.

| Population Name (Abbreviation) | Sample Size | Number of *cyt b* Haplotypes | Latitude of Population Center | Longitude of Population Center | Museum Catalog Numbers | GenBank Accession Numbers |
| --- | --- | --- | --- | --- | --- | --- |
| East Colorado (E_CO)* | 51 | 5 | \| 38.972 \| \| --- \| | -103.652 | NA | EU285613** (n=43), EU285616^#^ (n=5), EU285626, EU285617, EU285627 |
| Northwest Kansas (NW_KS)* | 3 | 2 | \| 39.297 \|  \| \| --- \| --- \| | -101.529 | FHSM 9045, 9046, 9222 | EU499422, EU499397, EU285616 |
| Northeast Kansas (NE_KS)* | 3 | 2 | \| 39.374 \|  \| \| --- \| --- \| | -95.105 | FHSM 9098; MVZ 234170, 234171 | EU499423, EU285613, EU499393 |
| Southwest Kansas (SW_KS)* | 6 | 5 | \| 37.378 \| \| --- \| | -100.907 | FHSM 8990, 8991, 8994, 8998, 9015, 9137 | EU285640, EU499420, EU499425, EU499399, EU285622, EU499429 |
| South Central Kansas (SCent_KS)* | 14 | 8 | \| 37.640 \|  \| \| --- \| --- \| | -98.740 | FHSM 8236, 8240, 8520, 8571, 9121-9123, 9134, 9143, 9145, 9147, 9148, 9150, 9151 | EU285621, EU285623, EU285630, EU285631, EU285633, EU285637, EU499398, EU499400, EU499401, EU499404, EU499418, EU499419, EU499421, EU499424 |
| Northwest Oklahoma (NW_OK)* | 7 | 3 | \| 35.913 \|  \| \| --- \| --- \| | -99.749 | OMNH 41706, 41707, 41708, 41709, 41710, 41711, 41712 | EU499434, EU499412, EU499435, EU499432, EU285634, EU499431, EU499406 |
| Central Oklahoma (Cent_OK)* | 10 | 7 | \| 35.617 \| \| --- \| | -97.540 | OMNH 40110; MVZ 145173-145177, 145205, 145206, 149680, 164812 | EU499433, EU499396, EU285617, EU499427, EU499430, EU285639, EU285628, EU499394, EU285632, EU499426 |
| East Central New Mexico (ECent_NM)* | 6 | 4 | \| 34.803 \| \| --- \| | -103.877 | NA | EU285613** (n=3), EU285619, EU285624, EU285635 |
| Texas Panhandle (TX_Pan)* | 22 | 5 | \| 33.62 \| \| --- \| | -100.84 | TNHC 60525, 60526 | EU285618, EU499416, EU499416** (n=11), EU285614 (n=1), EU285618*** (n=6), EU285636 (n=1), EU285638 (n=1) |
| West Texas (West_TX)* | 3 | 3 | \| 32.089 \| \| --- \| | -102.821 | TNHC 60528, 60529 | EU499415, EU285619, EU285614 |
| Southeast Arizona (SE_AZ)* | 64 | 6 | \| 31.82 \|  \| \| --- \| --- \| | -109.02 | NA | EU285615 (n=52), EU285620, EU285625, EU285629 (n=3), EU285641 (n=6), EU285642 |
| South Texas (S Texas) | 13 | 2 | \| 26.886 \|  \| \| --- \| --- \| | -98.254 | UTA A-60567, TNHC 94837 | OR256236 - OR256248 |

*Previously published *cytochrome b* sequences from Rice & Pfennig (2008)

**Rice & Pfennig (2008) provide the following accession numbers for this haplotype: EU285613, EU499393-EU499416

***Rice & Pfennig (2008) provide the following accession numbers for this haplotype: EU285618, EU499429-EU499435

^#^Rice & Pfennig (2008) provide the following accession numbers for this haplotype: EU285616, EU499418-EU499427
