# Supplementary material for: Population isolation in the Plains spadefoot toad: causes and conservation implications
Source: PeerJ. 2024 Oct 7;12:e17968. doi: 10.7717/peerj.17968 (PMC11466216; doi:10.7717/peerj.17968)
Supplement: Supplemental Information 2 [file peerj-12-17968-s002.docx]

| Species Name | N | Found in *Central Range*? | Found in region of proposed disjunction? | Found in *South Texas*? |
| --- | --- | --- | --- | --- |
| *Acris creptians* | 1769 | y | y | y |
| *Anaxryus woodhousii* | 2280 | y | y | n |
| *Anaxyrus punctatus* | 952 | y | y | y |
| *Anaxyrus speciosus* | 530 | y | y | y |
| *Bufo debilis* | 865 | y | y | y |
| *Craugastor augusti* | 14 | y | y | n |
| *Gastrophyrne carolinensis* | 198 | y | y | n |
| *Gastrophyrne olivacea* | 1672 | y | y | y |
| *Hyla chrysoscelis* | 986 | y | y | n |
| *Hypopachus variolosus* | 35 | n | y | y |
| *Pseudacris clarkii* | 810 | y | y | y |
| *Pseudacris streckeri* | 721 | y | y | n |
| *Rana areolata* | 145 | y | n | n |
| *Rana berlandieri* | 644 | y | y | y |
| *Rana catesbeiana* | 1028 | y | y | y |
| *Rana palustris* | 35 | n | y | y |
| *Rana sphenocephala* | 35 | n | y | y |
| *Scaphiopus couchii* | 1833 | y | y | y |
| *Scaphiopus hurterii* | 252 | y | y | y |
| *Spea multiplicata* | 1399 | y | y | n |
