# Supplementary material for: Population isolation in the Plains spadefoot toad: causes and conservation implications
Source: PeerJ. 2024 Oct 7;12:e17968. doi: 10.7717/peerj.17968 (PMC11466216; doi:10.7717/peerj.17968)
Supplement: Supplemental Information 3 — All models were replicated 10 times using cross-validation and the mean results were submitted. The two independent test samples came from the iNaturalist data and haplotype data as described in the main text. [file peerj-12-17968-s003.docx]

Supplemental Table 3. Model validation metrics. All models were replicated 10 times using cross-validation and the mean results were submitted. The two independent test samples came from the iNaturalist data and haplotype data as described in the main text.

| **Regularization multiplier** | **Mean Training AUC** | **Cloglog threshold (10% training presence)** | **Correctly predicted independent test file from iNaturalist** | **Correctly predicted independent test file from haplotype localities** |
| --- | --- | --- | --- | --- |
| 0.25 | 0.810 | 0.351 | 404/513 (78.8%) | 58/63  (92%) |
| 0.5 | 0.803 | 0.356 | 405/513  (78.9%) | 57/63  (90.5%) |
| 1 (full model) | 0.797 | 0.371 | 399/513 (77.8%) | 56/63  (88.9%) |
| 1 (reduced model) | 0.787 | 0.370 | 399/513  (77.8%) | 55/63  (87.3%) |
| 2 | 0.785 | 0.385 | 397/513 (77.4%) | 54/63  (85.7%) |
| 3 | 0.780 | 0.400 | 404/513 (78.8%) | 54/63  (85.7%) |
