# Supplementary figures and images for: Population isolation in the Plains spadefoot toad: causes and conservation implications
Source: PeerJ. 2024 Oct 7;12:e17968. doi: 10.7717/peerj.17968 (PMC11466216; doi:10.7717/peerj.17968)

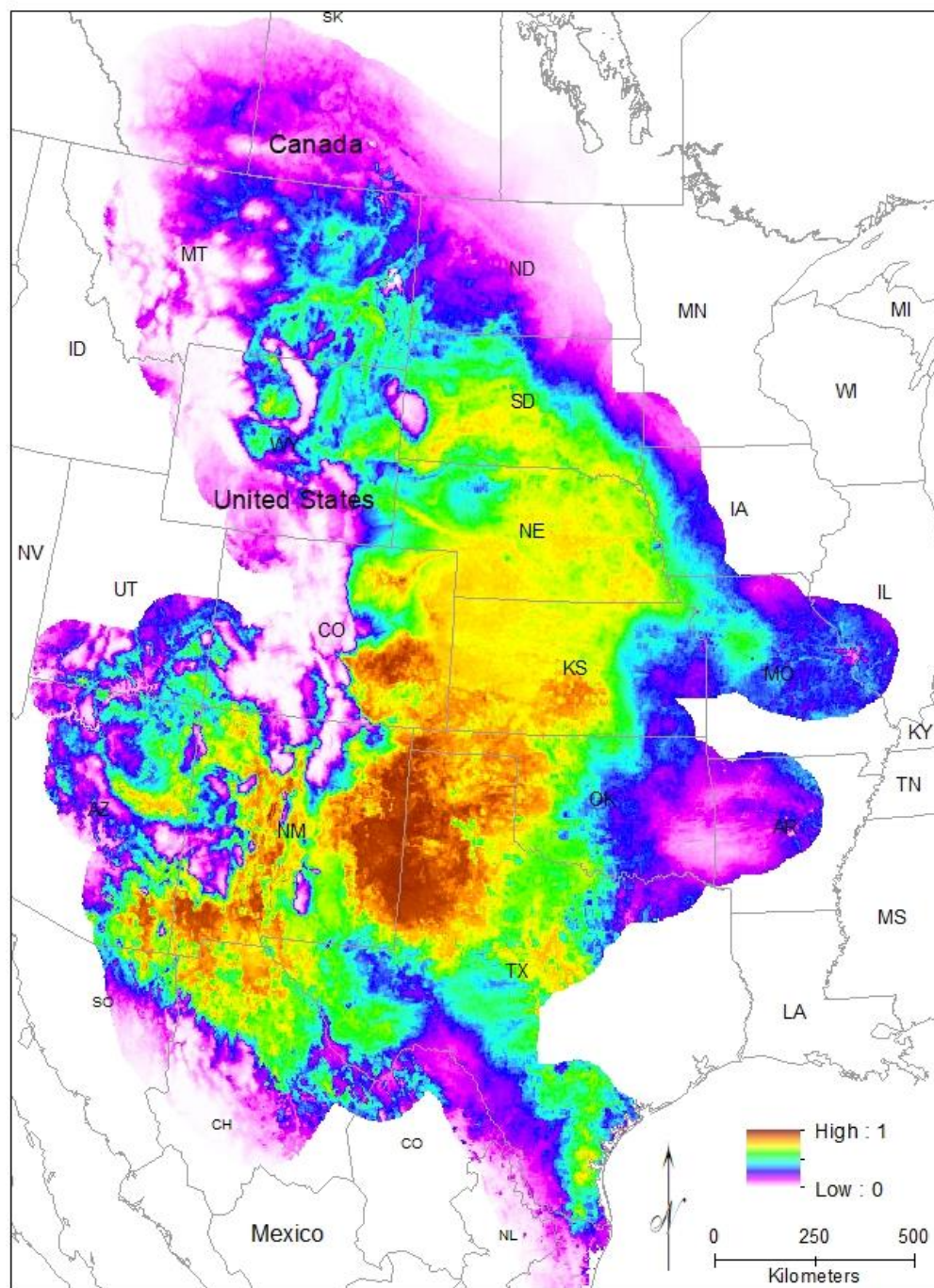

Supplement: Supplemental Information 4 — All localities and the default regularization multiplier (1.0) were used. [file peerj-12-17968-s004.pdf]
